# Roflumilast Suppresses Adipogenic Differentiation *via* AMPK Mediated Pathway

**DOI:** 10.3389/fendo.2021.662451

**Published:** 2021-06-07

**Authors:** Wan Xu, Jingjing Zhang, Jing Xiao

**Affiliations:** Department of Endocrine, Xiangyang Central Hospital, Affiliated Hospital of Hubei University of Arts and Science, Xiangyang City, China

**Keywords:** obesity, adipogenesis, Roflumilast, PPAR-γ, C/EBPα, AMPK

## Abstract

Obesity and related disorders have increasingly become global health problems over the years. In recent years, obesity has been recognized as the most important risk factor for a variety of diseases including cardiovascular diseases, type 2 diabetes, steatohepatitis, and cancer. The medical anti-obesity treatment is to intervene in the metabolic process of adipocytes by suppressing adipogenesis and promoting lipolysis. The Phosphodiesterase-4 (PDE4) pathway is involved in fat mass control and metabolic regulation. The present study aimed to investigate the effects of Roflumilast, a selective PDE4 inhibitor, on the differentiation of 3T3-L1 cells and the high fat diet-induced obesity in mice. We showed that treatment with Roflumilast inhibited lipid accumulation and triglycerides storage in mature 3T3-L1 cells, suggesting that Roflumilast suppressed adipogenesis. Mechanistically, we found that Roflumilast decreased the differentiation-induced expression of the adipogenesis genes including SREBP1C, FABP4, and Glut4, as well as their regulators including PPAR-γ and C/EBPα. Moreover, we proved that the effect of Roflumilast was dependent on the activation of the metabolic regulator AMPKα. The treatment with Roflumilast remarkably decreased the animals’ body weight, visceral adipose tissue weight, and adipocyte size in high fat diet-induced obese mice. In conclusion, our study revealed that Roflumilast suppressed adipogenesis and promoted lipolysis in cell culture and mice models *via* AMPK-mediated inhibition of PPAR-γ and C/EBPα. These findings imply roflumilast could have therapeutic potential in obesity-related diseases.

## Introduction

The prevalence of obesity has become a significant health burden in many countries. Driven mainly by unhealthy dietary habits and sedentary lifestyles, obesity is a significant risk factor for major chronic diseases, including type 2 diabetes and cardiovascular diseases (1). Adipose tissue is mainly made up of lipid-rich adipocytes, and it comprises about 20% of total body weight in healthy individuals. As the central metabolic organ, the main function of adipose tissue is to store energy and maintain homeostasis. Adipocytes store the energy as lipid droplets and secrete different adipokines to regulate energy homeostasis. In response to the oversupply of energy, adipocytes can rapidly expand their number and increase their size to store triglycerides (TG). The expansion of adipocytes is characterized by the differentiation of preadipocytes into mature adipocytes, a process called adipogenesis (2). However, the excessive energy storage in the adipose tissue often leads to pathological alteration and triggers pro-inflammatory responses of immune cells, which is an important pathological feature of obesity (3). The current intervention strategy for obesity is to balance the energy supply by preventing excessive uptake and deposition of fat. Therefore, agents that can inhibit adipogenesis are viewed as valuable in the treatment of obesity (4). Adipogenesis is regulated by many transcription factors and hormones. Peroxisome proliferator-activated receptor (PPAR) and CCAAT-enhancer-binding protein (C/EBP) families play major roles in adipocyte differentiation. Both PPARγ and C/EBPα are induced during the differentiation of preadipocytes. They also regulate each other and are essential to the differentiation process and the capacity to store lipids (5). Sterol regulatory element-binding transcription factor 1c (SREBP1c) is another key transcription factor that promotes adipogenic genes (6). Downregulating the activation of these pathways has been suggested as a potential treatment to prevent or reverse obesity (5).

**Figure 1:**
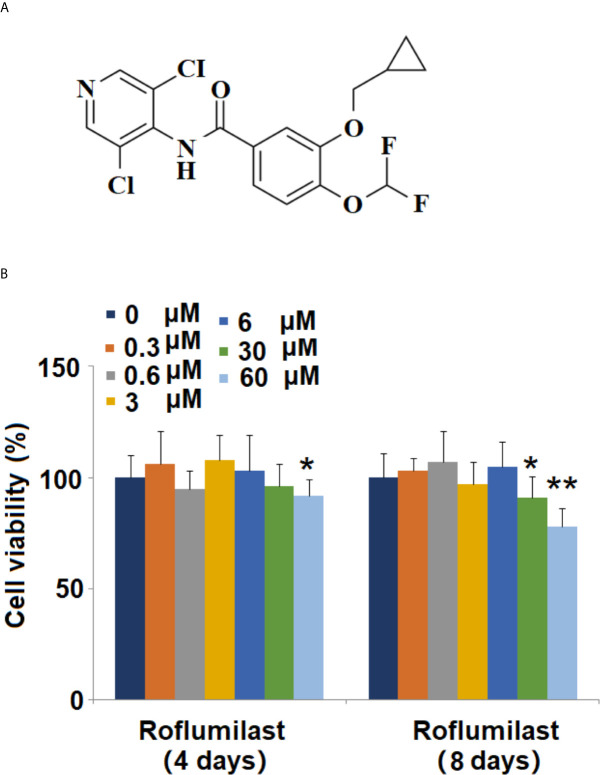
Effects of Roflumilast on cell viability of 3T3-L1 cells. Cells were stimulated with 0.3, 0.6, 3, 6, 30, and 60 μM Roflumilast for 4 and 8 days. **(A)** Molecular structure of Roflumilast; **(B)** Cell viability was determined at day 4 and 8 (*, **, P < 0.05, 0.01 *vs.* vehicle control, n = 6).

Phosphodiesterases 4 (PDE4) are a group of intracellular enzymes that degrade cyclic 3′,5′-adenosine monophosphate (cAMP), a second messenger in inflammatory cells. PDE4 inhibitors exhibit potent anti-inflammatory action and have been recognized as the novel therapeutic approach to treat severe chronic obstructive pulmonary disease (COPD) and asthma (7). Biological studies show that PDE4B-deficient mice have lower fat pad weights, and smaller adipocytes (8). Recent studies have demonstrated that the inhibition of PDE4 has therapeutic value for metabolic disorders related to obesity and type 2 diabetes (9–11). Roflumilast is a selective phosphodiesterase 4 (PDE-4) inhibitor, which was approved by the U.S. Food and Drug Administration (FDA) to treat COPD (12). The molecular structure of Roflumilast is shown in **Figure 1A**. Roflumilast exhibits a protective effect by suppressing airway inflammation and ROS production against various immune cells, such as neutrophils, pulmonary artery vascular smooth muscle cells, and airway epithelial cells (13). The preclinical study shows that Roflumilast reduces weight gain in obese mice (14). These data suggest Roflumilast-mediated PDE-4 inhibition may have certain regulatory effects in adipocytes. In the present study, we explored its molecular mechanism in adipocytes differentiation and evaluated its therapeutic value in high fat-induced obese mice.

## Materials and Methods

### Cell Culture and Adipogenic Differentiation

Mouse 3T3-L1 preadipocytes were purchased from American Type Culture Collection (ATCC). The cells were seeded in 12-well culture plates with complete medium (high glucose Dulbecco’s Modified Eagle Medium (Gibco), 10% fetal bovine serum (FBS), and 50 μg/ml penicillin/streptomycin). Upon confluence, the media were changed the following day and replaced with an adipogenic differentiation cocktail containing 0.5 mM 3-isobutyl-1-methylxanthine (IBMX), 1 mM dexamethasone (Sigma, USA). To test the drug cytotoxicity, the 3T3-L1 cells were exposed to 0.3, 0.6, 3, 6, 30, and 60 μM Roflumilast containing differentiation cocktail for 4 and 8 days. For all other treatment experiments, the cells were treated with 3 and 6 μM Roflumilast containing differentiation cocktail media for 8 days. The differentiation status was determined on day 8. Compound C was a specific inhibitor of AMPK, and 10-20 µM has been used to block AMPK activity in induction media treated 3T3-L1 cells ( (15). In this study, we used 10 µM concentration to block the activity of AMPK.

### MTT

3T3-L1 cells were plated in a 96-well plate at the density of 10, 000 cells per well and incubated overnight. Then the cells were fed with differentiation induction media containing roflumilast for 48 hours. 20 μl MTT solution (5 mg/ml) was added to the plate and incubated for 4 hours at 37 °. The formation of formazan product was dissolved by adding 200 μl DMSO. The plate was read at 492 nm on a microplate spectrophotometer (BioTek).

### Oil Red O Staining

Intracellular lipid content was visualized using Oil Red O staining (ORO, Sigma Chemicals). In brief, 3T3-L1 cells in a 96-well plate were differentiated as described before. On day 8, the cells were washed with PBS and fixed with 4% paraformaldehyde for 10 minutes, followed by rinsing with 60% isopropanol and staining with 0.21% Oil Red O (in 60% isopropanol) solution for 15 minutes. After washing the cells with distilled water 3 times, 100 µL of 100% isopropanol was added to extract the stain from the cells for 10 minutes. Finally, the absorbance was measure at 540 nm on a microplate reader (BioTek). The data were presented as fold change.

### Measurement of Triglycerides

The intracellular content of triglycerides (TG) of 3T3-L1 was measured with a commercial assay kit (Abcam). In brief, the cells in 12-well plates were washed with PBS, detached by trypsinization, and then resuspended in 1 ml homogenization solution. The resuspended cells were heated on a 100°C metal block for 2 minutes and cooled down at room temperature. The above step was repeated until the cells were completely lysed. The samples were cleared by centrifugation and subjected to assay procedure in a 96-well plate as described in the product’ manual. Finally, TG contents were read at OD 570 on a microplate spectrophotometer (BioTek).

### Measurement of Lipolysis

The assessment of lipolysis is the measurement of the lipolytic product glycerol released from adipocytes. In brief, the culture media of mature 3T3-L1 were collected on day 8. The content of glycerol released from the cells was detected using a commercial Free-Glycerol Reagent kit (Sigma). In brief, 20 µl of samples were reacted with 80 free glycerol reagents for 15 minutes at ambient temperature. The reacted samples were read at a wavelength of 540 nm on a microplate reader. The concentration of glycerol was normalized to a protein concentration of the sample.

### Real-Time PCR

Total RNA was extracted from 3T3-L1 cells using the RNeasy mini kit (Qiagen) by following the manufacturer’s manual. 1 μg purified RNA sample was used to synthesize cDNA. Reverse transcription was performed using the iScript II cDNA Synthesis Kits (BioRad). The expression levels of FABP4, GLUT4, SREBP-1c, PPARγ C/EBPa, and GAPDH were assessed by specific pairs reacted with Quantifast SYBR Green mix on a QIAquant 96 platform (Qiagen). PCR conditions were as follows: 95°C for 3 minutes, 40 cycles of 95°C for 10 s, 60°C for 30 seconds, and 72°C for 30 seconds, followed by 1 cycle of 95°C for 1 minute. GAPDH expression was used to normalize target gene expression. The 2^−ΔΔCt^ method was used to calculate the relative mRNA expression. GAPDH was used as a reference gene. The Forward and reverse primer sequences are listed in **Table 1**.

**Table 1:** Primer sequence.

Gene name	Forward primer	Reverse primer
FABP4	TGAAATCACCGCAGACGACAGG	GCTTGTCACCATCTCGTTTTCTC
GLUT4	GAGCCTGAATGCTAATGGAG	GAGAGAGAGCGTCCAATGTC
SREBP-1c	ATCGCAAACAAGCTGACCTG	AGATCCAGGTTTGAGGTGGG
PPAR γ	TTCAGCTCTGGGATGACCTT	CGAAGTTGGTGGGCCAGAAT
C/EBP a	GTGTGCACGTCTATGCTAAACCA	GCCGTTAGTGAAGAGTCTCAGTTTG
GAPDH	AAGAAGGTGGTGAAGCAGGCATC	CGAAGGTGGAAGAGTGGGAGTTG

### Western Blot Analysis

3T3-L1 cells on day 8 were collected and lysed in a RIPA buffer on ice. The whole-cell lysate was centrifuged at 14000 rpm for 10 minutes to obtain supernatant soluble protein. Protein concentration was measured using a DC Protein Assay kit (Bio-Rad). The same amount of 20 μg of each sample was separated on 10% SDS-polyacrylamide gel and transferred onto a nitrocellulose membrane (Millipore). The blots were then blocked with 5% non-fat dry milk for 1 hour and incubated overnight at 4°C with primary antibodies. After three times washing, the membranes were incubated with the secondary horseradish peroxidase-conjugated antibodies (Thermo Fisher Scientific, USA) for 1 hour. The amount of each protein was detected using an enhanced chemiluminescence assay kit (GE Healthcare).

### *In-Vivo* Experiments

The animal experiment protocols were approved by the Animal Care Committee of Affiliated Hospital of Hubei University of Arts and Science (HBUAS-ACC-20180016). 4-week-old male C57BL/6 mice were purchased from Jackson Laboratory (Bar Harbor, Maine). The mice were randomly allocated into 3 groups of 8 mice and treated as follows for 16 weeks: Normal group mice were fed a normal diet (11.4% fat); The HFD group mice were fed an HFD diet (60% fat); The HFD+ Roflumilast group mice were fed an HFD (60% fat) and administered orally every day by oral gavage (12.5, 25 mg/kg Roflumilast). The administered amounts of roflumilast were determined based on the doses used in previous studies (16, 17). The animals were housed on a 12/12-hour light/dark cycle and had free access to water and food ad libitum. At the end of the feeding cycle, the mice were euthanized by cervical dislocation to record the body weight and visceral tissue weight. The total visceral tissue weight was normalized to body weight as described in the previous publication (18).

### Statistical Analysis

All the results were presented as Mean ± S.E.M (standard error of mean) from at least three replicates. Statistically significant differences for continuous variables were determined using one-way analysis of variance (ANOVA) with Fisher’s least significant difference *post hoc* test. All testing was performed using GraphPad Prism 6 software. A p-value of less than 0.05 was considered statistically significant.

## Results

### Effects of Roflumilast on Cell Viability in 3T3-L1 Cells

The molecular structure of Roflumilast is shown in **Figure 1A**. To determine the cytotoxic effects on cell viability, 3T3-L1 cells were treated with different concentrations of Roflumilast, and the cell viability was measured using MTT assay. When the cells were treated for 4 days, only the highest dose of Roflumilast (60 μM) significantly decreased 8% of the cell viability (**Figure 1B**). When cells were treated for 8 days, the two highest doses (30, and 60 μM) resulted in about 9 and 22% reduction of cell viability, respectively (**Figure 1B**). Based on these data, 3 and 6 μM Roflumilast were both used in all other experiments.

### Roflumilast Inhibits Adipogenic Differentiation

**Figure 2:**
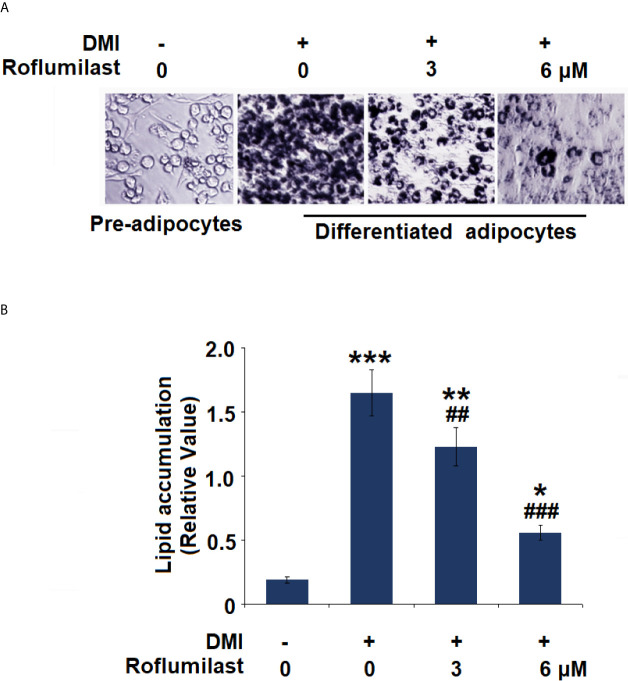
Effect of Roflumilast on adipogenesis in 3T3-L1 cells. Cells were incubated with differentiation cocktail (DMI) medium with Roflumilast (3 and 6 μM). **(A)** Cells were stained with Oil Red O on day 8; **(B)** Lipid accumulation was examined by measuring absorbance at 540 nm of Oil Red O (*, **, ***, P < 0.05, 0.01, 0.005 *vs.* vehicle group; ^##^, ^###^, P < 0.01, 0.005 *vs.* DMI group, n = 6).

To investigate the effect of Roflumilast on adipogenic differentiation, intracellular lipid accumulation was determined in mature adipocytes. Adipogenic differentiation of 3T3-L1 preadipocytes was examined on day 8 using Oil-Red O staining. Mature adipocytes were identified using the deposition of oil droplets. Based on microscopic observation, mature 3T3-L1 cells treated with Roflumilast maintained their fibroblastic shape and contained few lipid droplets (**Figure 2A**). Compared to undifferentiated cells, mature cells had a significant increase in lipid accumulation, which was remarkably rescued by the introduction of 3 and 6 μM Roflumilast, respectively (**Figure 2B**).

### Roflumilast Inhibits Triglycerides Deposition and Stimulates Lipolysis

**Figure 3:**
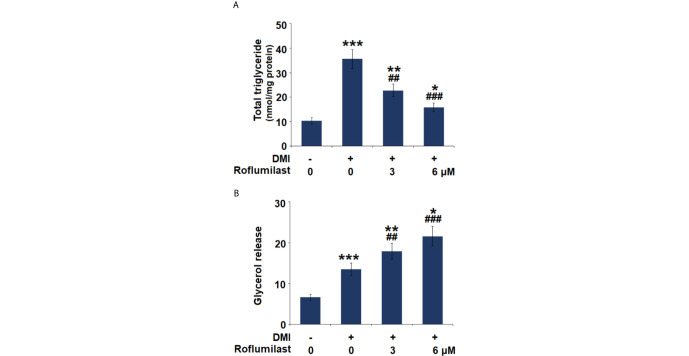
Effect of Roflumilast on triglycerides content and lipolysis. Cells were incubated with a differentiation cocktail (DMI) medium with Roflumilast (3 and 6 μM). **(A)** Total level of triglycerides; **(B)** Lipolysis is shown as glycerol release (*, **, ***, P < 0.05, 0.01, 0.005 *vs.* vehicle group; ^##^, ^###^, P < 0.01, 0.005 *vs.* DMI group, n = 6).

The intracellular content of triglycerides (TG) and glycerol release were also measured on day 8 of 3T3-L1 adipogenic differentiation. The mature cells stored more than 3.5-fold TGs, but treatment with 3 and 6 μM Roflumilast reduced lipid accumulation in cells to about 2.2- and 1.5-fold, respectively (**Figure 3A**). Meanwhile, the mature cells showed an approximately 2-fold increase in glycerol release, but the cells had about 2.7- and 3.2-fold increase in glycerol release in the presence of 3 and 6 μM roflumilast, respectively (**Figure 3B**). Therefore, Roflumilast treatment dose-responsively inhibited TG deposition but stimulated lipolysis in adipogenic differentiation.

### Effect of Roflumilast on the Expressions of Adipocyte-Specific Genes

**Figure 4:**
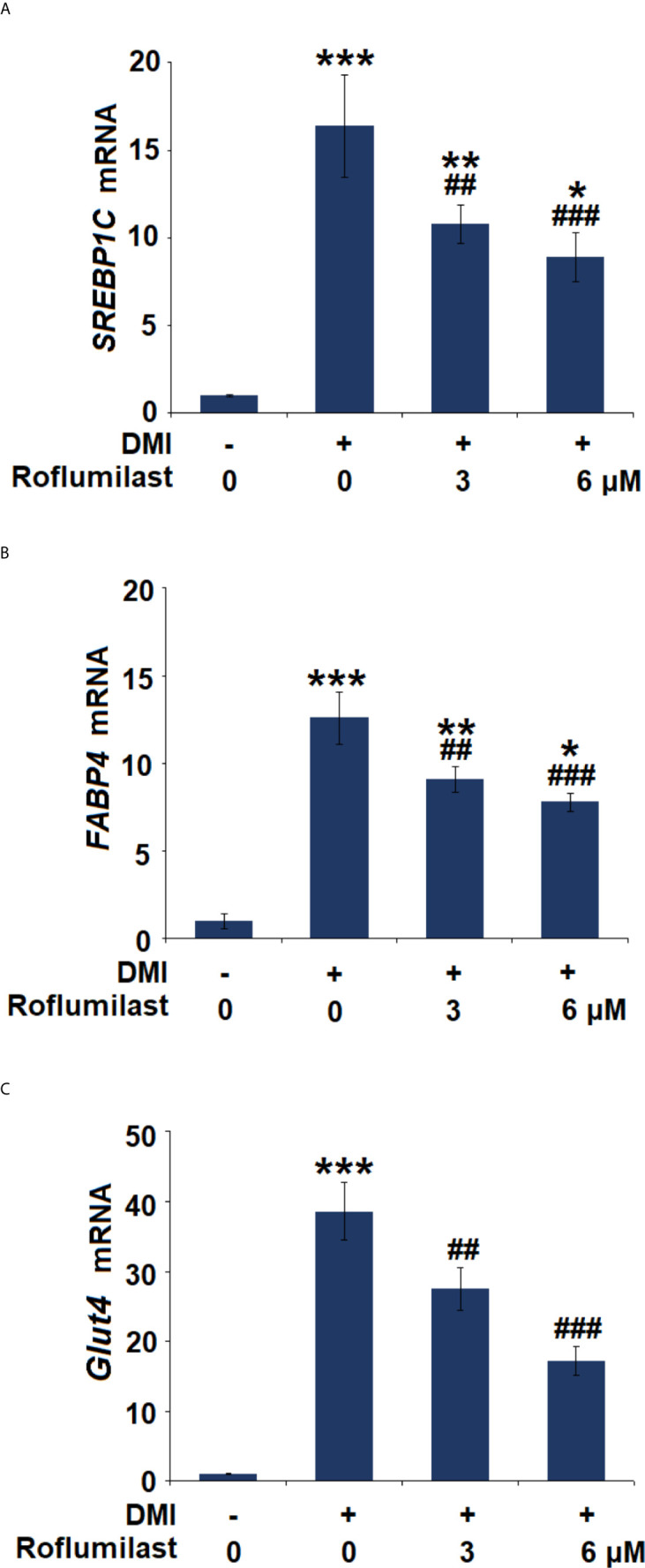
Effects of Roflumilast on the expression of adipogenic genes. Cells were incubated with a differentiation cocktail (DMI) medium with Roflumilast (3 and 6 μM). **(A)** mRNA of *SREBP1C;* **(B)** mRNA of *FABP4;* **(C)** mRNA of *Glut4* (*, **, ***, P < 0.05, 0.01, 0.005 *vs.* vehicle group; ^##^, ^###^, P < 0.01, 0.005 *vs.* DMI group, n = 6).

The differentiation of 3T3-L1 was complete about 7 days after treatment with the differentiation cocktail. During the process, several key molecules were induced. We then assessed the expressions of the adipocyte-specific genes, including SREBP-1c, FABP4, and Glut4. The differentiation cocktail induced 15.4-, 12.6-, and 38.6-fold increases in mRNA expressions of SREBP-1c (**Figure 4A**), FABP4 (**Figure 4B**), and Glut4 (**Figure 4C**), respectively. However, the increased trends in the mRNA levels of these genes in 3T3-L1 cells were massively attenuated by treatment with the two doses of Roflumilast. In the presence of 6 μM Roflumilast, the differentiation cocktail only induced about 7.9-, 6.8-, and 17.2-fold increases in the expressions of SREBP-1c (**Figure 4A**), FABP4 (**Figure 4B**), and Glut4 (**Figure 4C**), respectively.

### Effects of Roflumilast on the Expressions of Adipogenic Transcription Factors

**Figure 5:**
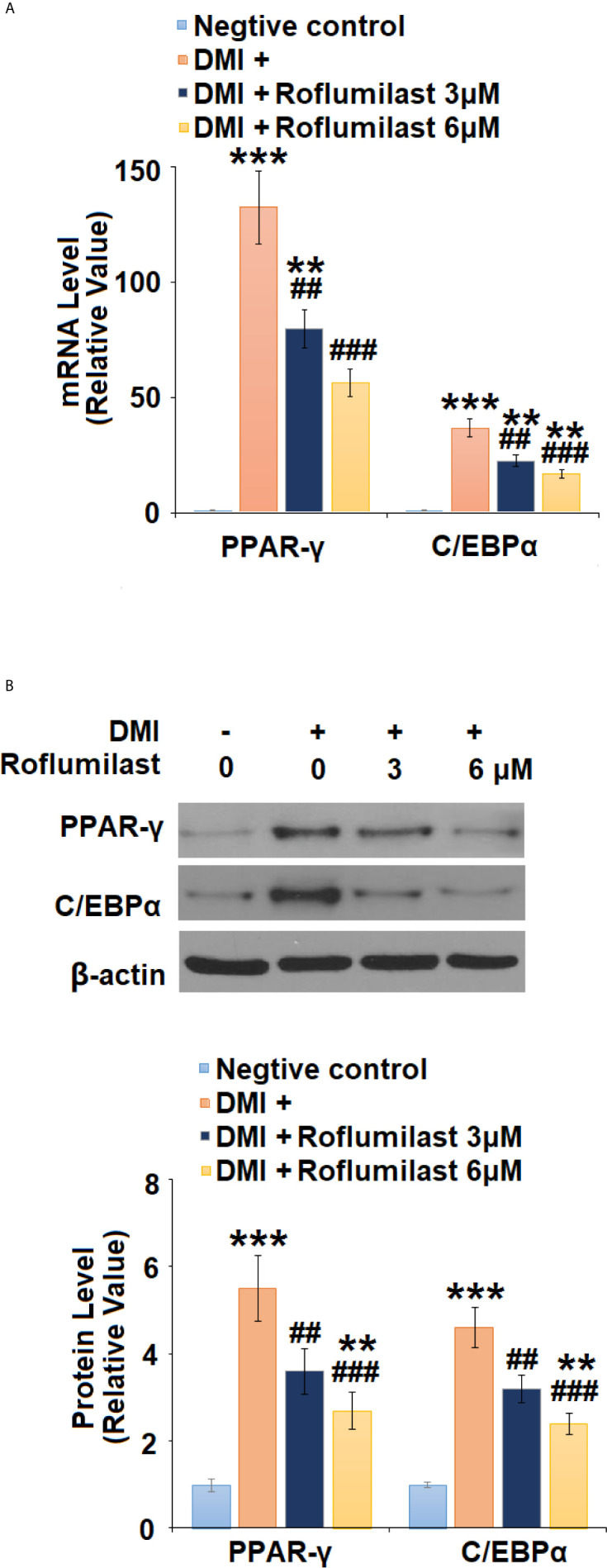
Roflumilast regulates adipogenic and lipogenic transcriptional factors. Cells were incubated with a differentiation cocktail (DMI) medium with Roflumilast (3 and 6 μM). **(A)** mRNA of PPAR-γ and C/EBPα; **(B)** Protein levels of PPAR-γ and C/EBPα, and the representative images of each protein were shown on the top panel, the quantification of protein level was shown on the bottom panel (**, ***, P < 0.05, 0.01, 0.005 *vs.* vehicle group; ^##^, ^###^, P < 0.01, 0.005 *vs.* DMI group, n = 4-5).

To understand the mechanism of roflumilast on Adipogenic differentiation, the expressions of two master adipogenic transcription factors, PPAR-γ and C/EBPα were investigated during adipogenic differentiation. At mRNA transcriptional level, the differentiation cocktail induced 132.5- and 12.6-fold increases in PPAR-γ and C/EBPα (**Figure 5A**) expressions, respectively. However, the introduction of the two doses of Roflumilast dose-responsively attenuated these increases, and the expression levels of PPAR-γ and C/EBPα (**Figure 5A**) were only increased to 56.3- and 16.9- fold, respectively. As for their protein levels, the differentiation induced 5.5- and 4.6-fold increases in PPAR-γ and C/EBPα protein levels (**Figure 5B**), respectively. However, the introduction of the two doses of Roflumilast again attenuated these increases, and the expression levels of PPAR-γ and C/EBPα (**Figure 5B**) were only increased to 2.7- and 2.4-fold, respectively.

### AMPK Pathway Mediates the Effects of Roflumilast

**Figure 6:**
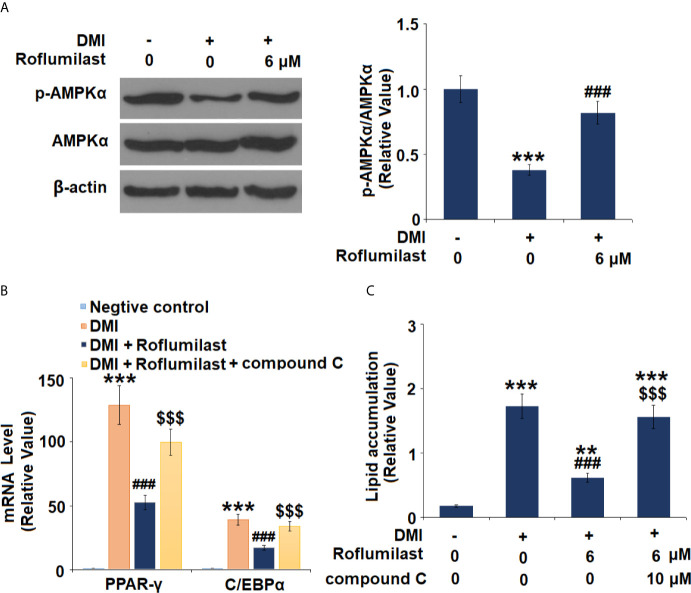
AMPKα mediates adipogenesis. **(A)** Cells were incubated with a differentiation cocktail (DMI) medium with Roflumilast (6 μM). Phosphorylated and total levels of AMPKα were measured, and the representative images of each protein were shown on the top panel, the quantification of p-AMPKα/AMPKα ratio was shown on the bottom panel; **(B)** Cells were incubated with a differentiation cocktail (DMI) medium with Roflumilast (6 μM) or the compound C (10 μM) for 8 days. mRNA of PPAR-γ and C/EBPα; **(C)** Lipid accumulation was examined by measuring absorbance at 540 nm of Oil Red O (**, P < 0.05 vs. vehicle group; ***, ^###^, ^$$$^, P < 0.005 *vs.* vehicle, DMI group, and DMI+ Roflumilast group, respectively, n = 4-5).

Next, the AMPK pathway was surveyed by detecting the activity of AMPKα in mature 3T3-L1 cells. 3T3-L1 treatment with differentiation cocktail repressed phosphorylation of AMPKα to 38%, but the co-treatment with 6 μM Roflumilast was able to recover 82% of AMPKα phosphorylation (**Figure 6A**). Furthermore, we found that blockage of AMPKα by compound C almost abolished the amelioration of Roflumilast on PPAR-γ and C/EBPα. When AMPKα is active, differentiation cocktail induced 130- and 39-fold high PPAR-γ and C/EBPα expression, while Roflumilast ameliorated the induction of PPAR-γ and C/EBPα to 53-fold and 17-fold, respectively. When AMPKα was inhibited, Roflumilast ameliorated the expressions of PPAR-γ and C/EBPα to 100-fold and 34-fold, respectively (**Figure 6B**). The Oil Red O staining showed that the differentiation cocktail increased about a 9.6-fold deposition of lipid in the cells. When AMPKα is active, Roflumilast treatment reduced lipid deposition to about 3.5-fold. When AMPKα was inhibited, Roflumilast treatment was almost abated, there was about 8.6-fold lipid deposited in the cells (**Figure 6C**).

### Roflumilast Attenuates High-Fat Diets- Induced Obesity in Mice

**Figure 7:**
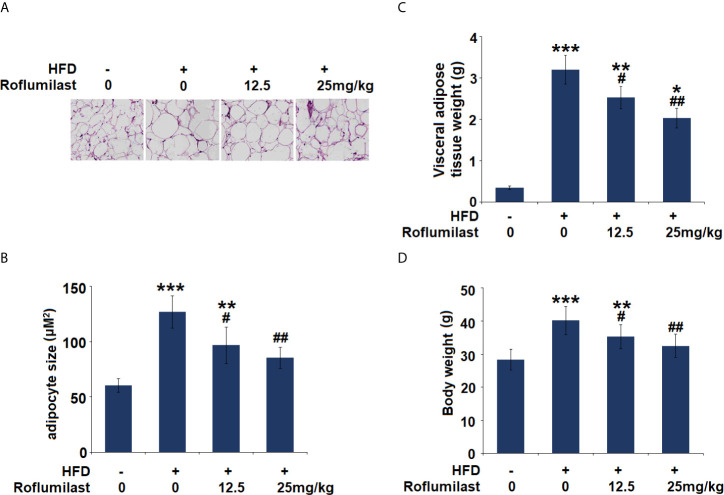
Effect of Roflumilast in HFD-induced obese mice. **(A)** Histological sections of visceral adipose tissue; **(B)** quantification of adipocyte size; **(C)** Visceral adipose tissue weight; **(D)** Bodyweight (*, **, ***, P < 0.05, 0.01, 0.005 *vs.* vehicle group; ^#^, ^##^, P < 0.05, 0.01 *vs.* HFD group, n = 8).

Finally, the effect of Roflumilast was tested in obese mice. In 16-week HFD-induced obese mice, synchronized Roflumilast treatment significantly reduced the fat pad (**Figure 7A**). Compared to non-obese mice, the size of adipocyte was 2.1-fold larger. While the size of adipocyte was about 1.6- and 1.4-fold large in two doses of Roflumilast treated mice (12.5, 25 mg/kg per day), respectively (**Figure 7B**). The high-fat diet feeding increased 9-fold weight of visceral fat tissue, but it only increased 7.2- and 5.8-fold increase of fat tissue in two doses of Roflumilast treated mice, respectively (**Figure 7C**). The high-fat diet-fed mice gained 40% more body weight, while the two doses of Roflumilast treated mice only gained about 25% and 15% more of body weight, respectively (**Figure 7D**).

## Discussion

The biological strategy to intervene with overweight and obesity is to suppress adipogenesis and increase the metabolism of adipocytes. Adipogenesis is the process of adipocyte differentiation from preadipocytes to mature adipocytes. Lipolysis is the hydrolytic reaction to the breakdown of triglycerides (TG) into fatty acids and glycerol. Thus, the effort to seek the agent that inhibits excessive adipogenesis and promotes lipolysis is an attractive strategy (19). In this study, we showed that the PDE-4 inhibitor Roflumilast could be a potential agent to possess this capacity. By incorporating Roflumilast into the induction medium, we found that its presence inhibited lipid deposition but promoted the release of glycerol during 3T3-L1 adipocytes differentiation, suggesting its dual action on adipogenesis and lipolysis.

**Figure 8:**
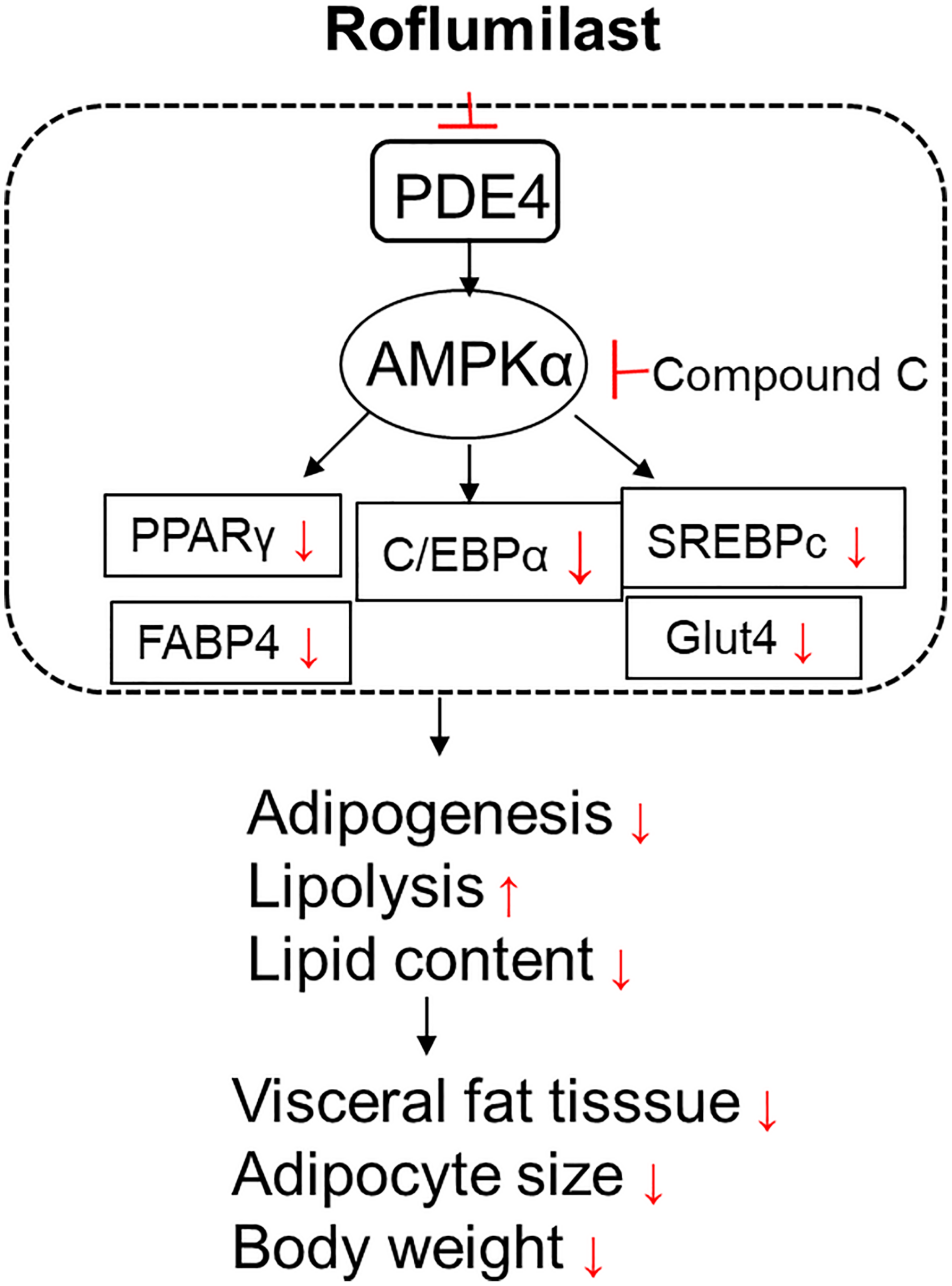
A graphic presentation of the underlying molecular mechanism.

Adipogenesis is controlled by several key transcriptional factors, including C/EBP family members and PPARγ, AMPK, and SREBPc. The expressions of PPARγ and C/EBPα in preadipocytes are undetectable at the initiation stage of adipocytes differentiation, but become detectable within 2 days of induction, and reach full expression within 5 days of induction (20). This activation of PPARγ and C/EBPα results in the expression of other specific factors that maintain the mature adipocyte phenotype. Adipogenesis is characterized by the accumulation of TGs and increases the activity of enzymes in lipolysis. Sterol regulatory element-binding proteins (SREBPc) are one of the late-stage factors controlling fatty acid synthesis and lipogenesis. AMPK was found to directly phosphorylate SREBP-1c and suppress the mature of SREBP-1c (21). AMP-activated protein kinase (AMPK) is a major regulator of cellular energy homeostasis. AMPK has a crucial role in the regulation of transcriptional factors and pathways related to adipogenesis and lipid synthesis (22). AMPK activation attenuated the expressions of PPARγ, C/EBPα, and SREBP-1c (23). As summarized in **Figure 8**, we showed that Roflumilast suppresses the expressions of adipocyte-specific genes SREBP-1c, FABP4, and Glut4. Mechanistically, Roflumilast suppressed the induction of expression but promoted AMPK activation. Importantly, the blockage of AMPK in 3T3-L1 differentiation abolished the inhibition of Roflumilast on PPARγ and C/EBPα, suggesting that AMPK activation is an essential upstream event in the Roflumilast-mediated suppression of adipogenesis.

To confirm our findings on the differentiation of 3T3-L1 cells, we administrated Roflumilast in the obese mouse model. The administration of Roflumilast for 16-weeks not only reduced the high fat diet-induced gain of body weight but also promoted fat mass loss. Interestingly, the Roflumilast-fed animals have a smaller adipocyte size, implying that the drug altered the lipid content of adipocytes. Hypertrophic adipocytes typically have an impaired cellular function, and the mechanisms to restrict their expansion and protect against their breakdown have been extensively investigated (24). The PDE-4 pathway was involved in metabolic regulation and weight gain (25, 26). Indeed, the administration of Roflumilast in mice promotes energy expenditure (14). In human subjects, the administration of Roflumilast promotes age-associated fat mass loss in people with metabolic syndrome (27) and weight loss in obese women (28). Although these data are from the small sample trials, it is encouraging that Roflumilast could influence the deposition of adipose tissue. Our study confirmed that the administration of Roflumilast regulates the metabolic status of adipocytes *in vitro* and *in vivo*, therefore the drug could have a potential role in the intervention of obesity.

The limitation of the study has to be addressed. Although we performed the experiments to verify the effect of Roflumilast in mice, only visceral adipose tissue and body weight parameters were assessed in this study. The target of Roflumilast could involve other cells other than adipocytes, and the mechanism of Roflumilast *in vivo* has not been understood completely. Obesity is a complex process involving many environmental and genetic factors (29). The modulation of Roflumilast on visceral obesity may exert direct actions on adipocytes or indirect action on other metabolic tissues. Recent findings indicate that Roflumilast could also regulate glucose levels by increasing energy expenditure in an animal experiment (14). Clinical trials have confirmed that Roflumilast has a robust regulation on glucose homoeostasis (30). The regulatory scope of Roflumilast covers several glucose control hormones, inflammatory markers, peripheral insulin sensitivity, and adiposity (27). In addition to adipocytes, the liver and muscles are also important organs that regulate glucose and lipid metabolism (31). The modulation of Roflumilast could be a pleiotropic effect involving multiple tissues. Our future study will provide a complete picture of the underlying mechanism.

In summary, our study shows that Roflumilast inhibits adipogenesis and enhances lipolysis in adipocyte induction *via* AMPK-mediated inhibition of PPAR-γ and C/EBPα. In high fat-induced obesity mice, the administration of Roflumilast decreases visceral fat deposition and body weight gain. These findings imply the PDE-4 inhibitor Roflumilast could have therapeutic application in obesity-related diseases.

## Data Availability Statement

The raw data supporting the conclusions of this article will be made available by the authors, without undue reservation.

## Ethics Statement

The animal study was reviewed and approved by Animal Care Committee of Affiliated Hospital of Hubei University of Arts and Science.

## Author Contributions

XW and ZJ conceived and designed the study. ZJ conducted the experiment. XJ contributed to the data analysis and wrote the manuscript. XW contributed to material and resources support. All authors contributed to the article and approved the submitted version.

## Conflict of Interest

The authors declare that the research was conducted in the absence of any commercial or financial relationships that could be construed as a potential conflict of interest.
